# P2X7 Receptor Regulates Collagen Expression in Human Intestinal Fibroblasts: Relevance in Intestinal Fibrosis

**DOI:** 10.3390/ijms241612936

**Published:** 2023-08-18

**Authors:** Lluis Lis-López, Cristina Bauset, Marta Seco-Cervera, Dulce Macias-Ceja, Francisco Navarro, Ángeles Álvarez, Juan Vicente Esplugues, Sara Calatayud, Maria Dolores Barrachina, Dolores Ortiz-Masià, Jesús Cosín-Roger

**Affiliations:** 1 Departamento de Farmacología, Facultad de Medicina, Universidad de Valencia, 46010 Valencia, Spain; lluislis@alumni.uv.es (L.L.-L.); cristina.bauset@uv.es (C.B.); macias.dcc@gmail.com (D.M.-C.); angeles.alvarez@uv.es (Á.Á.); juan.v.esplugues@uv.es (J.V.E.); sara.calatayud@uv.es (S.C.); dolores.barrachina@uv.es (M.D.B.); 2 FISABIO (Fundación para el Fomento de la Investigación Sanitaria y Biomédica), Hospital Dr. Peset, 46017 Valencia, Spain; marta.seco@uv.es; 3 Servicio Cirugía y Coloproctología, Hospital de Manises, 46940 Valencia, Spain; fran.navarro.vicente@gmail.com; 4 CIBERehd (Centro de Investigaciones en Red Enfermedad Hepática y Digestiva), 28029 Madrid, Spain; 5 Departamento de Medicina, Facultad de Medicina, Universidad de Valencia, 46010 Valencia, Spain; dolores.ortiz@uv.es

**Keywords:** P2X7 receptor, intestinal fibrosis, fibroblasts, Crohn’s Disease

## Abstract

Intestinal fibrosis is a common complication that affects more than 50% of Crohn´s Disease (CD) patients. There is no pharmacological treatment against this complication, with surgery being the only option. Due to the unknown role of P2X7 in intestinal fibrosis, we aim to analyze the relevance of this receptor in CD complications. Surgical resections from CD and non-Inflammatory Bowel Disease (IBD) patients were obtained. Intestinal fibrosis was induced with two different murine models: heterotopic transplant model and chronic-DSS colitis in wild-type and P2X7-/- mice. Human small intestine fibroblasts (HSIFs) were transfected with an siRNA against P2X7 and treated with TGF-β. A gene and protein expression of P2X7 receptor was significantly increased in CD compared to non-IBD patients. The lack of P2X7 in mice provoked an enhanced collagen deposition and increased expression of several profibrotic markers in both murine models of intestinal fibrosis. Furthermore, P2X7-/- mice exhibited a higher expression of proinflammatory cytokines and a lower expression of M2 macrophage markers. Moreover, the transient silencing of the P2X7 receptor in HSIFs significantly induced the expression of Col1a1 and potentiated the expression of Col4 and Col5a1 after TGF-β treatment. P2X7 regulates collagen expression in human intestinal fibroblasts, while the lack of this receptor aggravates intestinal fibrosis.

## 1. Introduction

Intestinal fibrosis represents one milestone in the management of Crohn’s Disease (CD) since approximately one half of CD patients will develop this complication, and more than 70% will need surgery during their lives [1]. In addition, a high degree of fibrosis recurrence has been found in intestinal surgeries, which are associated with an increased morbidity and reduced quality of life [2]. Therefore, it is necessary to develop new pharmacological drugs in order to treat and reverse this CD-associated complication. 

Intestinal fibrosis development is a long, slow, and progressive process based on the exacerbated accumulation of extra-cellular matrix (ECM) components and the expansion of the smooth muscle layers, which ends with intestinal obstruction [3]. Fibrotic strictures are originated due to a dysregulated mucosal healing joined with excessive chronic inflammation. Hence, both immune (such as macrophages or lymphocytes) and non-immune cells (including fibroblasts, myofibroblasts, and smooth muscle cells) play a crucial role in the fibrosis development [4]. Besides the specific role of different cell types in intestinal fibrosis, a wide range of molecules have been described as modulators of intestinal inflammation and fibrosis. Among all of them, extracellular purines have been identified as proinflammatory-signaling molecules that can regulate essential processes, such as cellular metabolism, proliferation, migration and apoptosis, in both autocrine and paracrine manners through activating P2Y and P2X receptors [5].

The P2X7 receptor is the unique member of the P2X receptor family, which differs structurally and functionally from the rest of the P2X subtypes. It can act as a detector of a danger signal because of its low ATP sensitivity [6]. Due to the fact that it is coupled with proinflammatory molecular cascades, for instance, the inflammasome assembly, it plays a crucial role in immunity and autoimmunity [7]. Growing evidence has directly described a proinflammatory role of the P2X7 receptor by activating the inflammasome pathway and releasing IL-1β [8,9,10]. Besides inflammation, P2X7R has also been linked with fibrosis development. Indeed, a profibrotic role of the P2X7 receptor has been described in the pathogenesis of a wide variety of inflammatory diseases related to the heart [11,12], liver [13,14], kidneys [15,16], and lungs [17,18] via different fibrotic and inflammatory mediators. In all these scenarios, it is suggested that the P2X7 blockade could attenuate fibrosis through various mechanisms, such as the reduction of inflammatory or fibrotic markers or the recruitment of inflammatory and immune cells modulating inflammation [19].

Specifically in the context of intestinal inflammation and fibrosis, there is accumulative literature describing the central role of P2X7R in the gastrointestinal inflammation. According to the literature, P2X7R increases the levels of proinflammatory mediators, mainly in immune cells such as macrophages or mast cells [20,21]. The expression of this receptor has been shown in several cell types of the colon that are involved in the pathogenesis of CD, such as mast cells, epithelial cells, T-cells, and enteric neurons [22]. Nevertheless, to our knowledge, the specific role of P2X7R in intestinal fibroblasts has not been deeply analyzed.

Therefore, in this study, we aim to analyze, for the first time, the role of P2X7R in the activation of primary human intestinal fibroblasts and the relevance of this receptor in intestinal fibrosis by using two different murine models. Here, we demonstrate that the absence of the P2X7 receptor worsens intestinal fibrosis development through the regulation of collagen expression in human primary intestinal fibroblasts.

## 2. Results

### 2.1. P2X7 Expression Is Increased in Intestinal Resections from CD Patients

**Figure 1:**
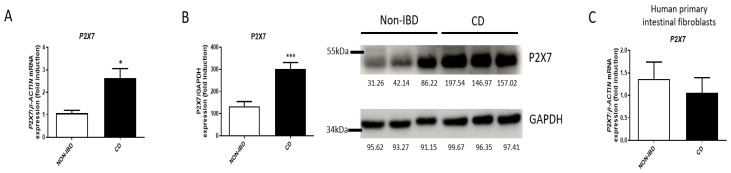
**P2X7 expression is increased in CD patients.** Intestinal surgical from CD patients and healthy tissue from colon carcinoma (non-IBD) patients were obtained. (**A**) Graph shows mRNA expression of P2X7 receptor in surgical resections from CD (*n* = 25) and non-IBD patients (*n* = 15). (**B**) Graph shows protein expression of P2X7 receptor in surgical resections from CD (*n* = 25) and non-IBD patients (*n* = 15). The data are expressed as fold induction in percentage. Representative image of a Western Blot showing 3 non-IBD and 3 CD patients. (**C**) Graph shows mRNA expression of P2X7 receptor in human primary intestinal fibroblasts isolated from surgical resections from CD (*n* = 5) and non-IBD patients (*n* = 5). Bars in graphs represent mean ± SEM. * *p* < 0.05 and *** *p* < 0.001 vs. non-IBD patients.

Firstly, we analyzed the expression of the P2X7 receptor in surgical resections from CD patients compared with healthy samples. mRNA analysis of this gene revealed a significant increase of the P2X7 receptor in CD surgical samples compared with control samples, as shown in Figure 1A. This result was reinforced when protein levels of P2X7 were also analyzed, and we detected a significant increase of the P2X7 receptor in CD samples compared with healthy samples (Figure 1B). In addition, we have also quantified the gene expression of the P2X7 receptor in human primary intestinal fibroblasts isolated from surgical resections from the same patients and, as shown in Figure 1C, non-significant differences were obtained between primary fibroblasts from CD patients versus those from control samples.

### 2.2. Lack of P2X7 Receptor Exacerbates Intestinal Fibrosis

Once the expression of the P2X7 receptor in human samples was analyzed, in order to determine the role of the P2X7 receptor, we established a murine model of intestinal fibrosis based on the heterotopic transplant model in both WT and P2X7 knock-out (P2X7-/-) mice.

**Figure 2:**
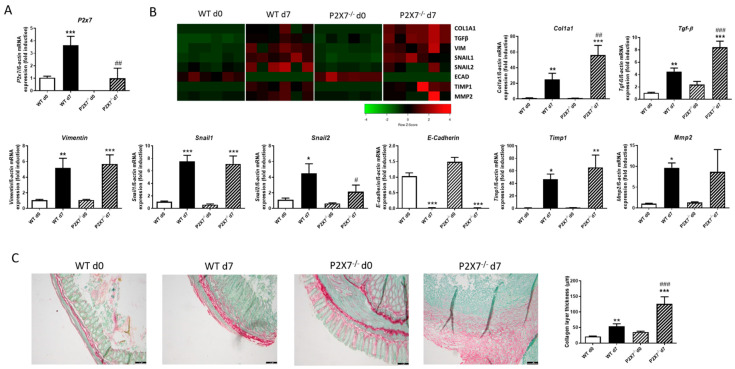
**Lack of P2X7 exacerbates intestinal fibrosis in murine heterotopic transplant model.** Murine intestinal fibrosis was induced with the heterotopic transplant model. (**A**) Graph shows mRNA expression of P2X7 receptor in intestinal grafts from both WT and P2X7-/- mice at Day 0 and Day 7 after surgery (*n* = 6). (**B**) Heat map showing the mRNA expression of profibrotic markers Col1a1, TGF-β, Vimentin, Snail1, Snail2, E-Cadherin, Timp1, and Mmp2. Graphs show the mRNA expression of these profibrotic markers in intestinal grafts from both WT and P2X7-/- mice at Day 0 and Day 7 after surgery (*n* = 6). (**C**) Representative images of intestinal grafts of the four experimental groups after Sirius Red Staining. Graph shows the quantification of the thickness of the collagen layer (*n* = 6). The length of the scale bar represents 100 µm. In all cases, bars in graphs represent mean ± SEM. * *p* < 0.05, ** *p* < 0.01, and *** *p* < 0.001 vs. intestinal graft from WT mice at Day 0; # *p* < 0.05, ## *p* < 0.01, and ### *p* < 0.001 vs. intestinal grafts from WT mice at Day 7.

In WT mice, the expression of the P2X7 receptor was significantly higher in intestinal grafts at Day 7 than those at Day 0 (Figure 2A). The expression of several profibrotic markers, such as, Col1a1, Tgf-β, Vimentin, Snail1, Snail2, and Timp1, was significantly increased, while that of E-Cadherin was absolutely reduced in grafts from both WT and P2X7-/- mice at Day 7 versus respective grafts at Day 0. Of interest, intestinal grafts from P2X7-/- mice 7 days after surgery displayed a significant increase in the expression of Col1a1 and Tgf-β, as well as a significant reduction in the expression of Snail2 compared with grafts from WT mice at Day 7. (Figure 2B). The Sirius Staining revealed that grafts at Day 7 presented a thicker collagen layer than the respective grafts at Day 0, whereas grafts from P2X7-/- at Day 7 exhibited a significantly thicker collagen layer than those detected in grafts from WT at Day 7 (Figure 2C).

**Figure 3:**
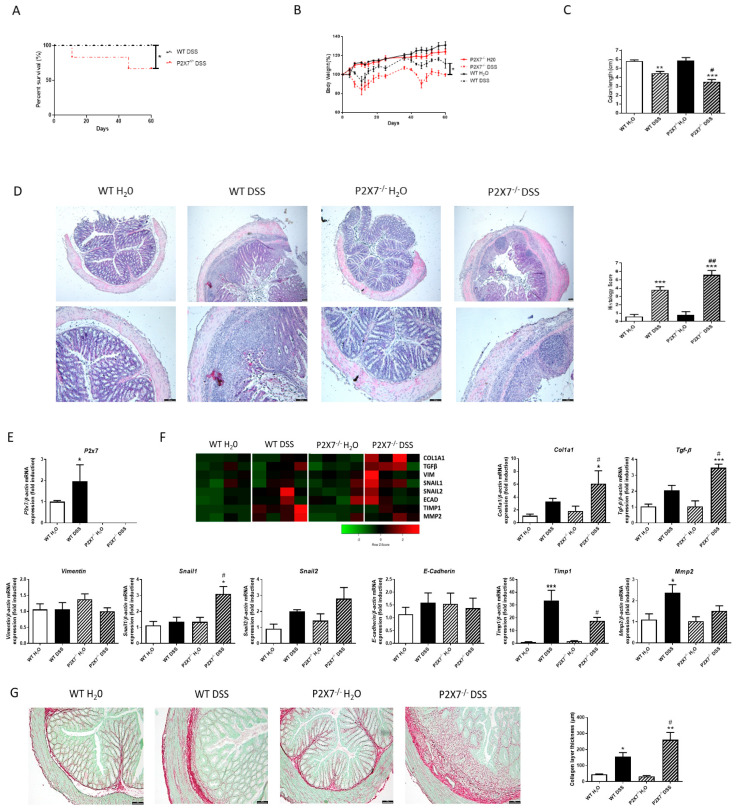
**Lack of P2X7 exacerbates intestinal fibrosis in chronic-DSS colitis model.** Chronic murine colitis was induced with four cycles of increasing percentages of DSS (1%, 1%, 1.5%, and 1.5%) in drinking water during 7 days, separated with 10 days for each cycle. (**A**) Graph shows the survival percentage in both WT and P2X7-/- mice after the four cycles of DSS (*n* = 5 per group). (**B**) Graph shows the evolution of body weight in each experimental group (*n* = 5 per group). (**C**) Graph shows the colon length of both WT and P2X7-/- mice with and without DSS (*n* = 5 per group). (**D**) Representative pictures of each experimental group after hematoxylin–Eosin staining (*n* = 5 per group). The length of the scale bar represents 100 µm. Graph shows the histological score obtained after the analysis of all the samples of each group. (**E**) Graph shows mRNA expression of P2X7 receptor (*n* = 5). (**F**) Heat map showing the mRNA expression of profibrotic markers Col1a1, TGF-β, Vimentin, Snail1, Snail2, E-Cadherin, Timp1, and Mmp2. Graphs show the mRNA expression of these profibrotic markers in intestinal samples from both WT and P2X7-/- mice with and without DSS (*n* = 4). (**G**) Representative images of intestinal samples from the four experimental groups after Sirius Red Staining. The length of the scale bar represents 100 µm. Graph shows the quantification of the thickness of the collagen layer (*n* = 4). In all cases, bars in graphs represent mean ± SEM. * *p* < 0.05, ** *p* < 0.01, and *** *p* < 0.001 vs. WT H_2_O; # *p* < 0.05 and ## *p* < 0.01 vs. WT–DSS mice.

As shown in Figure 3A, DSS administration induced a significant reduction in the percentage of survival of P2X7-/- mice, whereas it lacked any effect in WT mice. In parallel, DSS-treated P2X7-/- mice exhibited a higher loss of body weight and a significant reduction in colon length compared with DSS-treated WT mice (Figure 3B,C). In addition, the histological analysis shows more ulcerations and a higher loss of intestinal crypts in the colon of DSS–KO mice than that detected in DSS-treated WT mice (Figure 3D).

The expression of the P2X7 receptor was significantly increased in DSS–WT-treated mice compared with H_2_O–WT mice (Figure 3E). In this model, DSS–WT mice presented higher levels of Col1a1, Tgf-β, Snail2, Timp1, and Mmp2, which reached statistical significance in the last two genes. Of interest, P2X7-/--DSS-treated mice exhibited a significant increase in the expression of Col1a1, Tgf-β, and Snail1 compared with WT–DSS-treated mice, as well as a significant reduction in the expression of Timp1 (Figure 3F). The analysis of the collagen layer thickness also revealed that KO–DSS-treated mice showed a significantly thicker collagen layer than WT–DSS-treated mice (Figure 3G).

### 2.3. Lack of P2X7 Increases the Inflammatory Status and Alters the Macrophage Phenotype in Intestinal Mucosa

**Figure 4:**
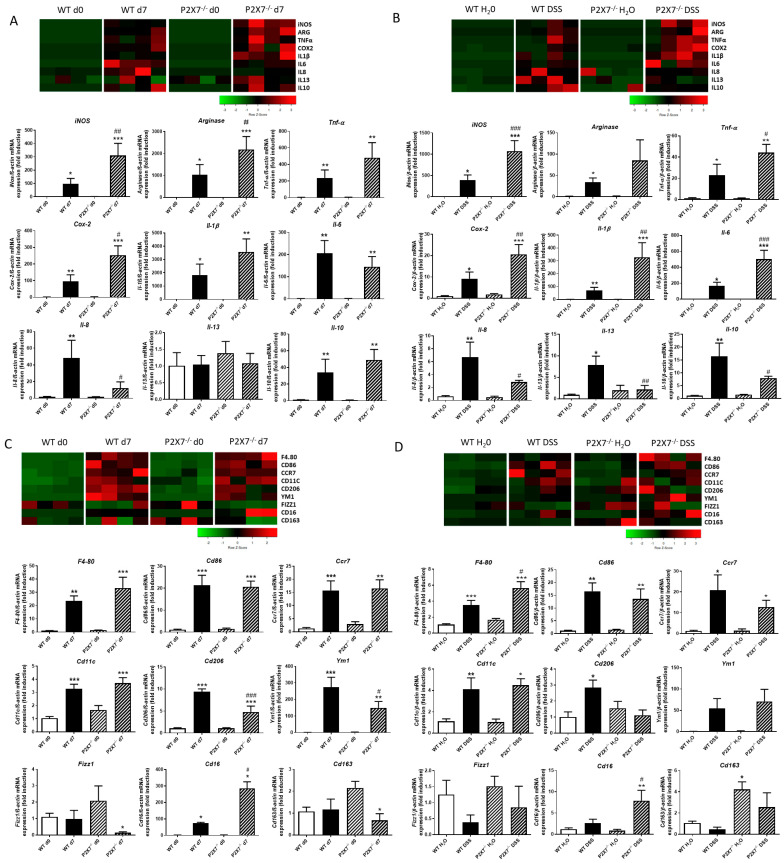
**Absence of P2X7 receptor alters the expression of proinflammatory, anti-inflammatory, and macrophage markers after murine intestinal fibrosis.** Murine intestinal fibrosis was induced by two different models: heterotopic transplant model and chronic administration of DSS in both WT and P2X7-/- mice. (**A**) Heat map showing the mRNA expression of iNOS, Arginase, Tnf-α, Cox-2, IL-1β, IL-6, IL-8, IL-13, and IL-10 after the heterotopic transplant model. Graphs show the mRNA expression of these genes in intestinal grafts from both WT and P2X7-/- mice at Day 0 and Day 7 after surgery (*n* = 4). (**B**) Heat map showing the mRNA expression of iNOS, Arginase, Tnf-α, Cox-2, IL-1β, IL-6, IL-8, IL-13, and IL-10 after the chronic administration of DSS. Graphs show the mRNA expression of these genes in both WT and P2X7-/- mice with and without DSS (*n* = 4). (**C**) Heat map showing the mRNA expression of F4/80, Cd86, Ccr7, Cd11c, Cd206, Ym1, Fizz1, Cd16, and Cd163 after the heterotopic transplant model. Graphs show the mRNA expression of these genes in intestinal grafts from both WT and P2X7-/- mice at Day 0 and Day 7 after surgery (*n* = 4). (**D**) Heat map showing the mRNA expression of F4/80, Cd86, Ccr7, Cd11c, Cd206, Ym1, Fizz1, Cd16, and Cd163 after the chronic administration of DSS. Graphs show the mRNA expression of these genes in both WT and P2X7-/- mice with and without DSS (*n* = 4). In all cases, bars in graphs represent mean ± SEM. * *p* < 0.05, ** *p* < 0.01, and *** *p* < 0.001 vs. WT Day 0 or WT H_2_O, depending on the murine model of intestinal fibrosis performed; # *p* < 0.05, ## *p* < 0.01 and ### *p* < 0.001 vs. WT Day 7 or WT–DSS mice, depending on the murine model of intestinal fibrosis performed.

As shown in Figure 4A, the analysis of cytokines and enzymes modulating inflammation revealed that intestinal grafts at Day 7 presented significantly higher levels of TNF-α, IL-1β, iNOS, IL-6, Arginase, Cox-2, IL8, and IL10 than those detected in grafts at Day 0, irrespective of the genetic background; among these genes, levels of iNOS, Arginase, and Cox-2 were significantly higher, while those of IL8 were lower in P2X7-/- than in WT mice. The absence of the P2X7 receptor does not significantly alter the basal mRNA expression of any of the above genes at Day 0.

However, WT–DSS-treated mice showed a significant increase in the expression of all cytokines and enzymes analyzed versus WT–H20 mice. Of interest, P2X7-/--DSS-treated mice exhibited a significant increased expression of iNOS, Tnf-α, Cox-2, IL-1β, and IL6 in parallel with a significant reduction of IL-8, IL-13, and IL10 compared with WT–DSS-treated mice (Figure 4B).

We next analyzed the expression level of the macrophage marker F4/80, which was significantly increased in intestinal grafts at Day 7 from both WT and P2X7-/- mice compared with their respective grafts at Day 0 (Figure 4C), as well as in both WT–DSS- and KO–DSS-treated mice (Figure 4D). A deeper analysis revealed an increased expression of the M1 markers Cd86, Ccr7, and Cd11c in both WT and P2X7-/- mice in both models with no differences between the genetic background of the mice. The analysis of M2 markers showed a significant increase in the expression of Cd206 in both experimental models of fibrosis in WT mice. However, in P2X7-/- mice, the expression of Cd206 was decreased in fibrotic tissue from both models, although it reached statistical significance only in the heterotopic transplant model. (Figure 4C,D). The absence of the P2X7 receptor caused a significant increase in Cd16 expression compared with that obtained in WT mice irrespective of the experimental model. (Figure 4C,D). Finally, we detected some differences in the expression of other M2 markers, such as Ym1, Fizz1, and Cd163, depending on the experimental model analyzed (Figure 4C,D).

### 2.4. P2X7 Regulates the Expression of Col1a1 in Human Intestinal Fibroblasts

**Figure 5:**
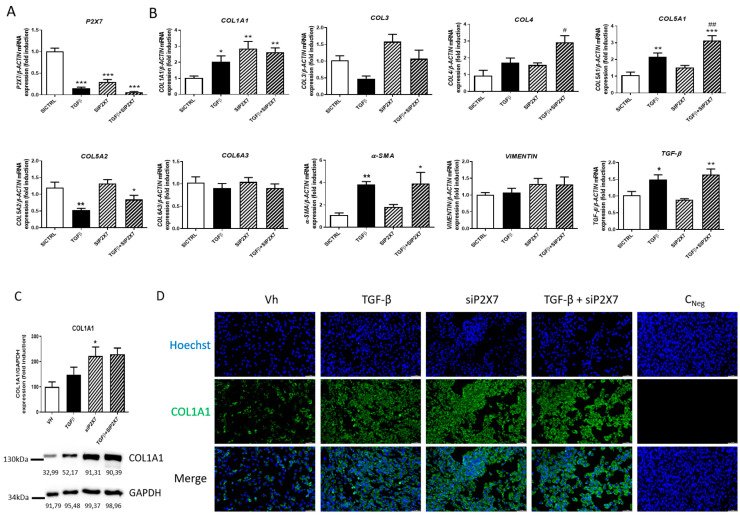
**P2X7 regulates collagen expression in HSIFs.** Primary human small intestine fibroblasts (HSIFs) were treated with TGF-β1 5 ng/mL during 24 h and transiently transfected with a specific siRNA against P2X7 receptor. (**A**) Graph shows mRNA expression of P2X7 receptor in HSIFs treated with TGF-β1 and with the P2X7 receptor expression transiently silenced (*n* = 5). (**B**) Graphs show the expression of COL1A1, COL3, COL4, COL5A1, COL5A2, COL6A3, α-SMA, VIMENTIN, and TGF-β in HISFs treated with TGF-β1 and transfected with a specific siRNA against P2X7 receptor (*n* = 5). (**C**) Graph shows protein levels of P2X7 receptor in HSIFs treated with TGF-β1 and with the P2X7 receptor expression transiently silenced (*n* = 5). The data are expressed as fold induction in percentage. (**D**) Representative pictures after immunofluorescence staining of COL1A1 in HSIFs treated with TGF-β1 and with the P2X7 receptor expression transiently silenced. The length of the white scale bar represents 50 µm. In all cases, bars in graphs represent mean ± SEM. * *p* < 0.05, ** *p* < 0.01, and *** *p* < 0.001 vs. siCtrl HSIFs; # *p* < 0.05 and ## *p* < 0.01 vs. TGF-β1-treated HISFs.

Finally, in order to elucidate the role of the P2X7 receptor in the expression of profibrotic markers, primary human intestinal fibroblasts (HSIFs) were transiently transfected with a specific siRNA against P2X7 and treated with TGF-β for 24 h. As shown in Figure 5A, treatment with TGF-β significantly reduced the expression of P2X7, while it induced a significant increase in the expression of COL1A1, COL5A1, α-SMA, and TGF-β compared with non-treated fibroblasts. In addition, the silencing of the P2X7 receptor caused a significant increase in the expression of COL1A1 in basal conditions, and it potentiated the increased expression of COL4 and COL5A1 in TGF-β-treated fibroblasts. However, the silencing of this receptor did not significantly modify the expression of COL3, COL5A2, COL6A3, α-SMA, or VIMENTIN (Figure 5B). Protein levels of COL1A1 were also quantified by western blot analysis, while HSIFs transfected with the specific siRNA against P2X7 showed significant increased levels of this protein compared with non-treated HSIFs (Figure 5C). These results were strongly reinforced with immunofluorescence experiments, which showed that treatment with TGF-β, the silencing of the P2X7 receptor, and the combination of both stimuli increased the protein levels of COL1A1 compared with non-transfected and non-treated intestinal fibroblasts (Figure 5D).

## 3. Discussion

The present study demonstrates for the first time that the lack of the P2X7 receptor exacerbates collagen deposition in two different murine models of intestinal fibrosis. The molecular mechanism behind this effect might be the direct regulation of collagen expression by this purinergic receptor in intestinal fibroblasts.

Our data show an increased mRNA and protein expression of P2X7 receptor in surgical resections from CD patients compared with control samples. These results are in line with Neves and colleagues, who reported increased levels of P2X7R in intestinal biopsies from both CD and UC patients compared with control samples [23]. In contrast to intestinal biopsies, surgical resections contain the whole thickness of the intestinal wall, while in complicated CD patients, an expansion of the mesenchymal compartment is frequently detected, which led us to further analyze the expression of the P2X7 receptor in isolated fibroblasts. Our results reveal no differences between those obtained from CD patients and control patients, which suggest that the increase detected in the whole tissue must be associated with other cells of the lamina propria, such as macrophages or lymphocytes, as previously reported [23].

Murine models of intestinal fibrosis are essential tools in order to better characterize the molecular mechanisms underlying this phenomenon. Although several models have been developed specifically for intestinal fibrosis, it is important to consider that each model induces fibrosis in a different manner [24]. To strengthen the conclusions of our study, we have chosen two different well-stablished murine models of intestinal fibrosis: the heterotopic transplant model and chronic–DSS administration [25,26,27]. In both, the induction of intestinal fibrosis was confirmed by the thicker collagen layer detected in the Sirius Red staining and the increased mRNA expression of several profibrotic markers. In accordance with the results obtained in surgical resections from CD patients, we found an increased expression of P2X7R in fibrotic colons from both models. Unexpectedly, P2X7-/- compared with WT mice exhibited a worsened intestinal fibrosis, characterized by a thicker collagen layer and a higher mRNA expression of Col1a1 and Tgf-β, which indicates an antifibrotic effect of P2X7R in the intestinal scenario. These results are in apparent contradiction with previous studies indicating a profibrotic role of P2X7R in different organs, such as the liver [28,29], kidneys [30], or the heart [11]. However, it is important to consider that cellular and molecular mechanisms involved in fibrosis might be different depending on the organ analyzed [31].

Inflammation is believed to be the main driver of fibrosis [32], and most studies analyzing fibrosis have linked the role of P2X7 to inflammasome activation [33] in immune cells such as macrophages [34] or neutrophils [28]. We detected, in WT mice, an increased expression of proinflammatory cytokines and macrophage markers in both experimental models of intestinal fibrosis. Of interest, results in P2X7-/- compared with WT mice exhibited a higher expression of both proinflammatory markers and the macrophage marker CD16, which has been shown to contribute to fibrosis in the intestine [33] and also in the liver [34] and skin [35]. In parallel, no differences in the expression of M1 markers, and a reduced expression of M2 marker CD206, were detected in P2X7-/- compared with WT mice. Our data suggest that P2X7, in intestinal inflammatory conditions, favors M2 polarization, which is reinforced by the reduced expression of IL13 and IL10, as well as the enhanced expression of proinflammatory cytokines detected in the colon of DSS-treated P2X7-/- mice. In line with our results, previous studies have shown that P2X7 promotes macrophage polarization towards a M2 phenotype in BMDMs [35] and alveolar macrophages [36]. If we look at the expression of proinflammatory cytokines, our results are in apparent contradiction with previous studies showing a proinflammatory role for this receptor in intestinal colitis. However, an important discrepancy is the fact that in these studies, the murine models of colitis were obtained in 7 or 8 days [23,37,38] and fibrosis was almost undetectable, while our experimental model consists of a 60-day model in which fibrosis is clearly established. Changes in cellular and molecular composition between fibrotic/non-fibrotic colon models or acute/chronic murine models might explain the differences observed.

Fibroblasts are the main components involved in extracellular matrix deposition; very little is known about the role of P2X7R in these cells. Here, we demonstrate for the first time that treatment with the profibrotic stimulus TGF-β induced a significant reduction in the expression of P2X7R in human small intestinal fibroblasts. In line with our results, previous studies reported that TGF-β treatment reduced the levels of P2X7R in the surface of peritoneal macrophages [39] and in THP-1 cells [40]. Furthermore, we found that the transient silencing of P2X7R with a specific siRNA increases both the basal expression of COL1A1 and the TGF-β-stimulated expression of COL4A1 and COL5A1 in these human primary intestinal fibroblasts. Intestinal fibrosis has been understood as an excessive wound healing [41]. It has been recently reported that the blockage of P2X7R in human dermal fibroblasts accelerated in vitro wound healing [42]. These results suggest that the excessive collagen accumulation detected in P2X7-/- compared with WT mice is related with the diminished levels of P2X7R in intestinal fibroblasts due to the increased expression of TGF-β1. However, considering the increased expression of this receptor detected in both murine and human fibrotic intestine, it seems likely that cells, other than fibroblasts, are involved in the upregulation of this receptor.

At this point, it is important to highlight the main limitations of this study that need to be considered. Firstly, the P2X7-/- mice used are complete knockouts, and all the cells of those mice lack the receptor. In order to better characterize the specific role of P2X7R in fibroblasts in intestinal inflammation and fibrosis, future experiments using conditional knockouts of P2X7R in fibroblasts should be performed in order to clarify this issue. Moreover, additional experiments with conditional knockout mice of P2X7R in macrophages will shed light on the specific role of the P2X7 receptor in macrophages and will show how these immune cells can impact intestinal fibrosis. However, the relevance of this receptor in human primary intestinal fibroblasts was analyzed, and we have demonstrated that the lack of this receptor increased col1a1 expression. Nevertheless, further experiments need to be performed in order to identify the molecular mechanisms involved in this novel regulation of *col1a1* by the P2X7 receptor.

Our study demonstrates in knockout mice that a chronic blockade of P2X7R might trigger a harmful consequence with even a higher degree of inflammation and intestinal fibrosis. This might explain the negative results obtained in clinical trials using a P2X7R antagonist in IBD patients. Indeed, the administration of the P2X7R antagonist AZD9056 in a Phase IIa study conducted in 34 CD patients showed only an improvement in the treatment of chronic abdominal pain but without any change in the inflammatory biomarkers, which questions the anti-inflammatory effect of blocking this receptor in a chronic pathological scenario [43]. Based on our results, we suggest that a blockade of this purinergic receptor in all cells, far from being beneficial, might alter intestinal fibroblasts, increasing their collagen deposition. Therefore, a better characterization of the cell-specific effect played by P2X7R in intestinal fibrosis is essential before planning the pharmacological modulation of this receptor in therapeutics. In addition, this mechanism should be considered in other fibrotic pathologies affecting the lung, liver, or heart, where a blockade of P2X7R in fibroblasts might also exert a harmful effect.

In summary, our results reveal an antifibrotic role of the P2X7 receptor in murine intestinal fibrosis. This effect is associated with the induction of anti-inflammatory cytokines and the collagen repression induced by this receptor in intestinal fibroblasts.

## 4. Materials and Methods

### 4.1. Patients

CD patients diagnosed according to Lennard–Jones’ criteria (clinical, biological, endoscopic, and histological) who required surgery were included in this study. Surgical resections from those patients were obtained. As control samples, healthy ileal surgical resections from patients with a colon carcinoma submitted to a surgery were obtained. In case of control patients (non-IBD), they were not undergoing chemotherapy before or at the time of surgery. The Institutional Review Board of the Hospital of Manises (Valencia, Spain) approved the study, following the Helsinki declaration recommendations. Written informed consent was obtained from all participating patients. The information related to the patients included in the study can be found in Table 1.

### 4.2. Isolation of Primary Human Intestinal Fibroblasts

Primary intestinal fibroblasts from surgical resections of both CD and non-IBD patients were obtained, as previously described [44]. Briefly, intestinal mucosa was cut into 3–5-mm pieces and incubated with HBSS–EDTA during 30 min at 37 °C in order to remove epithelial cells. Next, small mucosal pieces were digested with hyaluronidase (2 mg/mL), collagenase I (1 mg/mL), and DNAse (1 µL/mL) at 37 °C during 30 min. Then, intestinal samples were cultured in a Petri dish with DMEM high glucose (Sigma–Aldrich) supplemented with FCS 20%, penicillin/streptomycin (100 µg/mL), gentamycin (100 µg/mL), amphotericin B (2 µg/mL), and ciprofloxacin (16 µg/mL).

### 4.3. Mice

To perform in vivo experiments, wild-type (WT) C57Bl/6 and P2X7-/- mice (9–12 weeks old, 20–25 g weight) were bred. In all cases, mice were maintained under specific pathogen-free environment in the animal facility of the University of Valencia and were co-housed to reduce possible and potential effects of differences in microbiota. P2X7-/- mice, which were originally purchased from Jackson Laboratories (P2rx7 KO; B6.129P2-P2rx7^tm1Gab^/J) (Farmington, CT, USA), were kindly provided by Dra. Ángeles Álvarez Ribelles from University of Valencia. All protocols were approved by the institutional animal care and use committees of the University of Valencia, and all experiments were performed in compliance with the European Animal Research Laws (European Communities Council Directives 2010/63/EU, 90/219/EEC, Regulation (EC) No. 1946/2003), and Generalitat Valenciana (Artículo 31, Real Decreto 53/2013).

### 4.4. Induction of Intestinal Fibrosis

Intestinal fibrosis was induced in vivo using a heterotopic intestinal transplant model, as previously described [45]. In short, colon resections from WT and P2X7-/- donor mice sacrificed by neck dislocation were carefully washed with 0.9% NaCl to remove the stool and transplanted subcutaneously into the neck of WT-recipient mice. Therefore, recipient mice were anesthetized with isoflurane, and a small area of the back was shaved to avoid contamination with hair. Two subcutaneous incisions were made perpendicularly to the body axis, while the colon resections were implanted into each pocket and the skin was closed with vicryl 5-stiches. Seven days after the surgery, recipient mice were sacrificed by neck dislocation, and intestinal grafts were collected. A colon segment from each donor mouse was kept so it could be used as autologous control tissue (referred to as Day 0).

### 4.5. Induction of Chronic DSS Colitis

WT and P2X7-/- mice received vehicle or Dextran Sulfate Sodium (DSS, 40 kDa, Sigma-Aldrich, St. Louis, MO, USA) with four cycles of increasing percentages of DSS (1%, 1%, 1.5%, and 1.5%) in the drinking water solution during 7 days, intercalated by 10 days with water. Hence, there were a total of four groups: WT vh, WT DSS, P2X7-/- vh, and P2X7-/- DSS (*n* = 5 mice per group).

Body weight and clinical signs of disease were monitored from Day 1. At the end of the last cycle, on Day 60, mice were properly sacrificed by neck dislocation, and colon tissue samples were obtained for further analysis.

### 4.6. Cell Culture

To perform in vitro experiments, human small intestine fibroblast (HSIF) cells (Innoprot, Bizkaia, Spain) were cultured. Fibroblast medium (Innoprot, Bizkaia, Spain) supplemented with penicillin/streptomycin, fibroblast growth supplement, and inactivated fetal bovine serum was used. Depending on the experiment, HSIF cells were treated for 24 h with TGFβ 5 ng/mL.

### 4.7. Small Interfering (siRNA) Transfection

HSIF cells were transfected using a specific P2X7 siRNA (Ambion, Foster City, CA, USA) at a concentration of 20 pmol with Lipofectamine RNAiMAX (Invitrogen Life Technologies, Barcelona, Spain) following the manufacturer’s instructions. As a control of transfection, an siRNA (siCtrl) was used. Gene expression of P2X7 was analyzed by qPCR in order to determine the efficiency of the transfection.

### 4.8. Protein Extraction and Western Blot Analysis

Protein was isolated from HSIF cells, as previously described [46]. To analyze protein expression, Western Blot was performed. SDS–PAGE gels were used, and proteins were loaded at the same amounts. Then, proteins were transferred to nitrocellulose membranes and incubated with specific primary antibodies (detailed in Table 2), as well as with the secondary antibody peroxidase-conjugated anti-rabbit IgG (Invitrogen, Waltham, MA, USA, A16096, 1:5000). Protein bands were detected with Immobilon^®^ Crescendo Western HRP Substrate (Millipore, Burlington, MA, USA) in AMERSHAM ImageQuant 800 (GE lifescience, Cornellà de Llobregat, Spain). Glyceraldehyde 3-phosphate dehydrogenase (GAPDH) was used as housekeeping to normalize protein bands, whose densitometry was quantified by Multi Gauge V3.0 software (Fujifilm Life Sciences, Cambridge, MA, USA).

### 4.9. RNA Isolation and Real-Time Quantitative PCR (RT–qPCR)

RNA isolation was performed using direct-zol RNA MiniPrep Plus R2072 from ZymoResearch according to the manufacturer’s instructions. Mice colon samples were homogenized with TRI Reagent^®^ (ZymoResearch, Irvine, CA, USA) using the GentleMACS Dissociator (Milteny Biotech, Gladbach, Germany), as previously described [25]. HSIF samples were lysed with the lysis buffer provided by the kit and a 25 G needle. After RNA isolation, it was quantified with Nanodrop. In order to obtain cDNA, a reverse transcription PCR was performed using the the PrimeScript RT reagent Kit (Takara Biotechnology, Dalian, China). Gene expression was analyzed by real-time Quantitative PCR using SYBR^®^ Ex Taq (Takara Bio Inc., Saint-Germain-en-Laye, France) in LightCycler thermocycler (Roche Diagnostics, Mannheim, Germany). Table 3 and Table 4 show designed, specific oligonucleotides to perform the analysis. The relative gene expression, as fold increase, was expressed as follows: change in expression (fold) = 2 − Δ(ΔCT) where ΔCT = CT (target) − CT (housekeeping) and Δ(ΔCT) = ΔCT (treated) − ΔCT (control), where β-actin was the housekeeping gene used.

### 4.10. Hematoxylin–Eosin Staining

To analyze histology after the induction of both models of murine intestinal fibrosis, mice colonic tissues were paraffin-embedded in 5-µm sections and stained with Hematoxylin–Eosin. Slides were deparaffinized, rehydrated, and then incubated with Hematoxylin 1:4 (Sigma–Aldrich, Madrid, Spain) during 4 min at room temperature. Then, ethanol–HCl 0.5% and ammonium hydroxide 1% were added. Finally, aqueous eosin Y solution (Sigma–Aldrich, Madrid, Spain) diluted with glacial acetic acid 0.5% was added for 2 min at room temperature, dehydrated, and visualized with a light microscope (Leica DMi8, L’Hospitalet de Llobregat, Spain) using LEICA LAS X software version 5.1. The parameters of Obermeier et al. [47] were used to analyze the histology of the tissue. Briefly, as detailed in Table 5, it consisted of a scale from 0 to 8, which represented: (i) The presence of erosion; (ii) The depth and surface extension lesions in the epithelium; and (iii) The degree of inflammatory infiltrate. The total histological score represented the sum of the epithelium and infiltration score (total score = E + I).

### 4.11. Sirius Red Staining

To analyze the thickness of collagen layer, mice colonic tissues were paraffin-embedded in 5-µm sections and stained with Sirius Red. Slides were deparaffinized, rehydrated, and then incubated with Fast Green (Sigma–Aldrich, Madrid, Spain) during 15 min at room temperature and with Sirius Red 0.1% (Sigma–Aldrich, Madrid, Spain) and Fast Green 0.04% for 30 min at room temperature. Finally, slides were dehydrated and collagen layer, represented by red coloration, was observed with a light microscope (Leica DMDMi8, L’Hospitalet de Llobregat, Spain) using LEICA LAS X software and measured its thickness using ImageJ (National Institutes of Health, Bethesda, MD, USA). The measurement was performed in a blinded manner by an observer unaware of the corresponding group for each mouse.

### 4.12. Immunofluorescence

HSIF cells were fixed with 2% paraformaldehyde over 20 min and permeabilized with 0.1% Triton–X100 for 10 min. Next, cells were sequentially incubated with blocking solution (PBS with 10% serum and 1% BSA) at room temperature for 1 h, with the primary antibody anti-Collagen 1 (1:100, Abcam ab34710) at 4 °C overnight, and the secondary antibody (anti-rabbit-IgG-FITC, 1:1000, Southern Biotech, Birmingham, AL, USA) for 45 min at room temperature. Moreover, all nuclei were stained with Hoechst33342 (2 µM) during 5 min. Finally, cells were visualized with the microscope Leica DMDMi8. Images were captured with the software LASX Office 1.4.5 (Leica Application Suite X).

### 4.13. Statistical Analysis

Data were expressed as mean ± SEM and were compared by a *t*-test for comparisons between two groups and by analysis of variance (one-way ANOVA) with Tukey post hoc correction, or Kruskal–Wallis with Dunn’s post-hoc correction where suitable for multiple comparisons. Statistical significance was considered with a *p*-value < 0.05.

## Figures and Tables

**Table 1:** Clinical information of patients analyzed in the study.

		CD	Non-IBD
**Number of patients**		25	15
Age	Mean	47	66
	Interval	(30–70)	(37–80)
Gender	Male	9	6
	Female	16	9
CD phenotype	B2 or stricturing	15	-
	B3 or penetrant	10	-
Concomitant Medication	Azathioprine	16	-
	Methotrexate	5	-
	6-Mercaptopurine	3	-
	Biological Therapy (anti-TNFα)	24	-

**Table 2:** Primary antibodies used for Western Blot analysis.

Antibody	Supplier	Dilution (μL)
COLLAGEN 1	Ab34710; Abcam	1:1000
P2X7	Ab93354; Abcam	1:1000
GAPDH	G9545-200UL; Sigma	1:15,000

**Table 3:** Sequences of human primers used in real-time PCR.

Gene	Sense (5′–3′)	Antisense (3′–5′)	Fragment’s Size (bp)
*a-SMA*	GCTTCCCTGAACACCACCCA	ACAGAGCCCAGAGCCATTGT	131
*COL1A1*	GGAGCAGACGGGAGTTTCTC	CCGTTCTGTACGCAGGTGAT	252
*COL3A1*	CGCCCTCCTAATGGTCAAGG	TTCTGAGGACCAGTAGGGCA	161
*COL4A1*	CCGGATCACATTGACATGAAACC	TGGAAACCAGTCCATGCTCG	236
*COL5A1*	CCTGACAAGAAGTCCGAAGGG	GCGTCCACATAGGAGAGCAG	107
*COL5A2*	TGGTGCTGAAAGAAGAGCCC	TCTGACAAGGGGCAGGTTTC	281
*COL6A3*	GGCCGTCTTTTGCCTCTTTC	TGTTCCTCTCCAATGGTCCAAG	132
*P2X7*	TGCCGAAAACTTCACTGTGC	ATGCCCATTATTCCGCCCTG	209
*TGF B*	AGCAACAATTCCTGGCGATAC	CGGTAGTGAACCCGTTGATG	202
*VIMENTIN*	ATGAAGGAGGAAATGGCTCGTC	GGGTATCAACCAGAGGGAGTGAA	196

**Table 4:** Sequences of mouse primers used in real-time PCR.

Gene	Sense (5′–3′)	Antisense (3′–5′)	Fragment’s Size (bp)
*Arg*	GTGGGGAAAGCCAATGAAGAG	TCAGGAGAAAGGACACAGGTTG	232
*Ccr7*	CTCTCCACCGCCTTTCCTG	ACCTTTCCCCTACCTTTTTATTCCC	125
*Cd11c*	TCTTCTGCTGTTGGGGTTTG	CAGTTGCCTGTGTGATAGCC	204
*Cd16*	GAAGGGGAAACCATCACGCT	GCAAACAGGAGGCACATCAC	293
*Cd206*	TGTGGAGCAGATGGAAGGTC	TGTCGTAGTCAGTGGTGGTTC	201
*Cd86*	GCACGGACTTGAACAACCAG	CCTTTGTAAATGGGCACGGC	194
*Col1a1*	CAGGCTGGTGTGATGGGATT	AAACCTCTCTCGCCTCTTGC	317
*Cox-2*	CCCGGACTGGATTCTATGGTG	TTCGCAGGAAGGGGATGTTG	153
*E-cadherine*	AACCCAAGCACGTATCAGGG	ACTGCTGGTCAGGATCGTTG	142
*F480*	TGTCTGAAGATTCTCAAAACATGGA	TGGAACACCACAAGAAAGTGC	211
*Fizz1*	CGTGGAGAATAAGGTCAAGGAAC	CAACGAGTAAGCACAGGCAG	212
*Il10*	GGACAACATACTGCTAACCGAC	CCTGGGGCATCACTTCTACC	110
*Il13*	GCCAAGATCTGTGTCTCTCCC	ACTCCATACCATGCTGCCG	106
*Il1b*	TGCCACCTTTTGACAGTGATG	ATGTGCTGCTGCGAGATTTG	136
*Il6*	GAGTCCTTCAGAGAGATACAGAAAC	TGGTCTTGGTCCTTAGCCAC	150
*Il8*	CTGCTGGCTGTCCTTAACC	TCTGTTGCAGTAAATGGTCTCG	150
*iNos*	CGCTTGGGTCTTGTTCACTC	GGTCATCTTGTATTGTTGGGCTG	222
*Mmp2*	GCCAACTACAACTTCTTCCCC	CAAAAGCATCATCCACGGTTTC	112
*P2x7*	TAGGTGAGGGTTTGCTGTGG	ATGCCTTTGACCTTGGTGTG	281
*Snail1*	ATGCACATCCGAAGCCACAC	GGTCAGCAAAAGCACGGTTG	148
*Snail2*	GAAGCCCAACTACAGCGAAC	ATAGGGCTGTATGCTCCCGA	123
*Tgf-β*	GCGGACTACTATGCTAAAGAGG	TCAAAAGACAGCCACTCAGG	295
*Tnf-a*	GATCGGTCCCCAAAGGGATG	GGTGGTTTGTGAGTGTGAGGG	86
*Vimentin*	GCTCCTACGATTCACAGCCA	CGTGTGGACGTGGTCACATA	190
*Ym1*	AGAAGCAATCCTGAAGACACC	GCATTCCAGCAAAGGCATAG	205

**Table 5:** Histological score parameters.

	Epithelium (E)		Infiltration (I)
0	Normal morphology	0	No infiltrate
1	Loss of epithelial cells	1	Infiltrate around crypt basis
2	Loss of epithelial cell in large areas	2	Infiltrate reaching to *L. muscularis* mocusae
3	Loss of crypts	3	Extensive infiltration reaching the *L. muscularis* mucosae and thickening of the mucosa with abundant oedema
4	Loss of crypts in large areas	4	Infiltration of the *L. submucosa*
